# Crust treatments to reduce bread staling

**DOI:** 10.1016/j.crfs.2021.03.004

**Published:** 2021-03-27

**Authors:** Yi Chen, Theodoros Gavaliatsis, Simon Kuster, Christian Städeli, Peter Fischer, Erich J. Windhab

**Affiliations:** a ETH Zurich, Institute of Food, Nutrition and Health, Schmelzbergstrasse 7, 8092, Zürich, Switzerland; b Jowa AG, Erlenwiesenstrasse 9, 8604, Volketswil, Switzerland

**Keywords:** Bread, Crust, Coatings, Crust modifications

## Abstract

Crust treatments, namely edible bread coatings, enzymatic crust modification and chemical crust modification, were introduced with the intention to minimize bread water loss during ambient storage. It was observed that compared to the treated bread, the untreated bread had significantly higher weight loss and crumb firmness after 14 days of ambient storage. A large array of materials was tested, among which hydrophobic coatings were shown to have the highest moisture barrier efficiency. In particular, the 20% candelilla wax coating (solution of 20% candelilla wax in sunflower oil), 20% beeswax coating (solution of 20% beeswax in sunflower oil) and HPMC oleogel coating (coating containing hydroxypropyl methyl cellulose oleogel) were proved to be most effective, thanks to their low affinity with water and low water vapor permeability. The application of the 20% candelilla wax coating resulted in reductions of the bread weight loss from about 30 to 13% and the crumb firmness from above 500 to 34 ​N after a storage period of 14 days. In addition, it was noted that the enzymatic and chemical crust modifications yielded moderately good results, but showed a significantly altered appearance of the bread crust.

## Highlights

• Wax coating as edible food coating is used to inhibit bread staling.
• Moisture barrier by the wax coating is applied to the bread crust.
• Coatings significantly minimize the bread weight loss and crust hardening.
• Crust morphology is monitored by micro-CT.

## Graphical abstract

**Figure:**
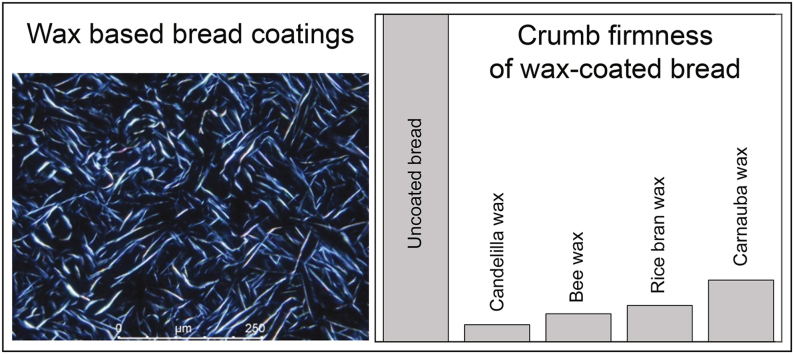

## 1 Introduction

Crust is the outer part of the bread formed during baking, which is comprised of a network of denatured gluten proteins and partially gelatinized starch granules (Altamirano-Fortoul et al., 2015). The fast evaporation of water from the crust during baking impairs the full gelatinization of starch granules in the crust, resulting in a lower degree of gelatinization, which is responsible for the particular material properties of the crust (e.g. glass transition, water sorption, crispiness, etc.) (Primo-Martin et al., 2007). Fresh crust is described as dry, crispy and brittle with an audible fracture (Primo-Martin et al., 2006). During storage, crust becomes leathery and loses its crispiness, which is considered as crust staling and is believed to be caused by moisture transfer from the crumb to the crust, leading to increases in water content and water activity (Altamirano-Fortoul et al., 2015; Gray and Bemiller, 2003). At room temperature, proteins and incompletely gelatinized starch molecules in the fresh crust are in glassy state. With the absorption of water, the crust undergoes a glass to rubber transition, which leads to a loss of the crispy behavior (Primo-Martin et al., 2006). This can be explained by the fact that water acts as a plasticizer and the rise in the water content decreases the glass transition temperature of the materials in the crust (Altamirano-Fortoul et al., 2015).

Edible coatings are thin layers of edible materials applied to a product surface, which provides barriers to moisture, oxygen, and solute movement (Bourtoom, 2008). Edible coatings are widely used in fresh fruits and vegetables to maximize their quality and shelf life by reducing moisture and solute migration, gas exchange, respiration and oxidative reaction rates (Dhall, 2013). Generally speaking, edible coatings should possess suitable mechanical properties and hydrophobic properties to exert low permeability to oxygen and water vapors. The commonly used coating materials are polysaccharides, proteins, resins, lipids and their combinations (Marelli et al., 2016). Among them, polysaccharides are most widely investigated and are known to form coatings with mechanical properties which are suitable for film forming and application. The coatings formed with polysaccharides demonstrate effective barrier properties to gas transport (O_2_, CO_2_), but with a high water vapor permeability (Bertuzzi et al., 2007; Falguera et al., 2011). Proteins are also suitable materials for edible coatings owing to their excellent mechanical and structural properties. However, they are commonly hydrophilic and susceptible to moisture absorption (Dhall, 2013). On the other hand, lipids can improve moisture barrier properties of coatings but they normally demonstrate poor mechanical strength (Falguera et al., 2011).

Edible coatings have been applied and patented for improving the quality of bakery products (Fadda et al., 2014). Glazing is one of the finishing procedures, which is applied for the surface coating on baked products to improve their flavors and appearance (Jahromi et al., 2012). Furthermore, a study from Johromi et al. (Jahromi et al., 2012) showed that certain glazes could act as a moisture barrier therefore increased crumb moisture and decreased crumb hardness during storage with respect to the control. Likewise, Chin et al. (2011) applied cornstarch, egg white, skim milk and shortening as glazes on bread surfaces and investigated their ability to reduce bread staling. They revealed that the application of cornstarch, egg white and skim milk was effective in reducing crumb firmness and in retaining some crumb moisture content (about 1%) during a storage period of 6 days.

The use of wax-based coatings is a relatively new idea and such coatings exhibit excellent moisture barrier properties (Dhall, 2013). Wax is an ester of a long-chain aliphatic acid with a long-chain aliphatic alcohol, which is insoluble in water (Lee et al., 2005). Doan et al. (2018) stated that adding waxes has proven to be most efficient in structuring liquid oil into a three-dimensional network due to their high oil-binding capacities. Wax-based coatings are effective in blocking transport of moisture thanks to their low polarity. However, waxes tend to form thick and brittle films (Quiros-Sauceda et al., 2014). Waxes such as beeswax and candelilla wax have also been applied in different types of meat in order to reduce moisture loss and to improve the outer surface appearance. They are considered fit to be eaten when applied in a thin layer (Hassan et al., 2018). In addition, Yilmaz and Dagdemir (2012) applied a beeswax coating on Kashar cheese and found out that the beeswax-coated cheese had a lower moisture loss. They also discovered that the beeswax-coated cheese presented a decrease of 2.5 logarithmic units on mold counts compared to the control at the end of 120 days storage period. It is worth mentioning that waxes such as beeswax, candelilla wax and carnauba wax are used in confectionery products such as candy to preserve individual piece identity as well as prevent moisture absorption or loss (Weller et al., 1998; Embuscado, 2009). However, there is few literature on the application of wax-based coatings on bread in order to reduce moisture loss and to retard bread staling.

In this work, the main goal of crust treatments was to modify the barrier properties of the bread crust with the intention of reducing and delaying the moisture loss of bread during ambient storage. Various crust treatments, namely crust coatings, enzymatic crust modifications and chemical crust modifications were investigated. The purpose of applying crust coatings was to provide a physical water resistant barrier to reduce water loss. The crust modifications were deployed to alter the crust microstructure, making it harder for water to pass through. Regarding the chemical modification, sodium hydroxide was applied with the aim to modify the gluten network in the crust and the crust properties. With respect to the enzymatic modification, amylase was chosen to alter the microstructure of crust. The application of edible coatings is one of the common approaches to control moisture transfer in foods. Therefore, a wide range of edible coating ingredients, including waxes, gum, starch, protein and cellulose derivatives were studied. In order to compare the efficacy of different treatments, the weight loss and the textural property (i.e. crumb firmness) of the bread samples over time were examined. Edible coatings are frequently reported to be applied in fruits and vegetables, with the aim to reduce water loss. However, only few studies have been conducted to investigate on the effects of coatings to reduce bread moisture loss and crumb hardening during storage. This research opens the possibility of applying coatings and other crust treatments (i.e. enzymatic modifications and chemical modifications) to extend bread shelf life.

## 2 Material and methods

### 2.1 Materials

Wheat flour (white flour, type 550) was procured from Stadtmühle Schenk AG. Baker’s yeast (Presshefe 100138), manufactured by Hefe Schweiz AG was used. Non-iodized table salt obtained from Schweizer Rheinsalinen AG and regular tap water were used. Candelilla wax, beeswax (yellow) and rice bran wax were obtained from Koster Keunen Holland. Carnauba wax (primagelb) and tragacanth gum powder were supplied by C. E. Roeper GmbH. High-oleic sunflower oil was purchased from Florin AG. Ethylcellulose Ethylcellulose (ethoxyl content 48%, 10 ​mPas) was from Acros Organics. Sodium hydroxide, corn zein and xanthan gum (xanthomonas campestris) were provided by Sigma Aldrich. α-amylase (*Bacillus subtilis*) was from MP Biomedicals. Hydroxypropyl Methyl Cellulose (HPMC) was offered by DOW Chemicals. Starch (Sobex 222) was from Südstärke GmbH.

### 2.2 Bread preparation

Bread samples were baked in accordance to a recipe composed of 1000 ​g wheat flour, 25 ​g yeast, 22 ​g salt, and 616 ​g water. A dough of 5 ​kg was prepared using a kneading machine (IRIS-030, Tekrom, Italy). Subsequently, 3 ​kg of dough was taken out from the kneading machine and flattened to a thickness of approximately 18 ​mm with the help of a sheeter (Type 2446, Seewer Rondo, Switzerland). Afterwards, the flattened dough was placed into a rounding machine (Type 130312, Divider Rounder DR Robot2 Automatic White-Blue, Daub Bakery Machine B.V., Netherlands). Thereafter, the rounded dough loaves (100 ​± ​5 ​g) were stored in a freezer at around - 18 ​°C.

### 2.3 Preparation of coating

The concentration of 20% (w/w) was chosen for the wax coatings. 20 g of each wax was melted in 80 ​g of high-oleic sunflower oil respectively on a magnetic stirrer (Heidolph Typ MR, 2002 N° 50304) at a temperature of approximately 150 ​°C. Similarly, 20% (w/w) ethylcellulose coating was acquired by melting 20 ​g ethylcellulose in 80 ​g high-oleic sunflower oil on the magnetic stirrer at around 250 ​°C until the mixture became transparent and homogenous. 50% (w/w) corn zein coating solution was obtained by dissolving zein in ethanol (94% aq). 1% (w/w) tragacanth solution was made by adding tragancanth in distilled water at room temperature. 20% (w/w) starch-based coating (sobex 222) was prepared in 85 ​°C water bath. The HPMC oleogel coating was prepared according to the method described by Patel et al. (2014): 0.8% (w/w) HPMC was added in 38.6% (w/w) distilled water. Afterwards, 60% (w/w) high-oleic sunflower oil was dispersed in the HPMC solution using a high energy dispersing unit (Polytron PT 6000, Kinematica GmbH, Switzerland) at a rotation speed of 3000 ​rpm, followed by the addition of 0.6% (w/w) xanthan gum solution under continuous shear. Subsequently, the emulsion was dried at 90 ​°C in an oven (UF 110plus, Memmert GmbH, Germany) for 48 ​h in order to remove water. Prior to the application on the bread, the HPMC oleogel was heated on the magnetic stirrer (stirrer was set to a temperature around 150 ​°C).

### 2.4 Coating of bread samples

To prepare the coated bread samples, some dough loaves were taken out from the freezer and placed on the baking trays. The loaves were then thawed at room temperature for 1 ​h. After thawing, the loaves were then inserted into a proofing chamber (Type GS20ED, Wiesheu GmbH, Germany) for 30 ​min at 32 ​°C with a relative humidity of approximately 80%. Subsequently, the proofed loaves were placed into a preheated (225 ​°C) industrial oven (Dibas 64, Wiesheu GmbH, Germany) with an injection of 250 ​ml steam, followed by a baking time of 10 ​min at 210 ​°C. Right after baking, the bread rolls were removed from the oven. The coatings were immediately applied on the hot breads with a commercial kitchen brush. Then the coated bread rolls were left to cool for around 2–3 ​h.

### 2.5 Crust treatments

To prepare the breads samples with crust treatments, some dough loaves were taken out from the freezer and thawed at room temperature for 1 ​h. After thawing, 4.9% (w/w) sodium hydroxide in distilled water and 10% (w/w) α-amylase in distilled water were sprayed onto the whole surface of the thawed loaves respectively right before putting them into the proofing chamber and subsequently baking oven (the proofing and baking conditions were the same as described in previous section).

### 2.6 Storage of bread samples

The bread samples were packed individually in paper bags (250 ​g, PAWI Verpackungen AG, Switzerland). The packed bread samples were then stored in a climate chamber (Kälte 3000, Switzerland) over a period of 14 days. The storage conditions were set to a temperature of 23 ​°C and a relative humidity of 65%. The humid environment (higher than the water activity of crust, which is approximately 0.8) was not considered in the study. When bread is exposed to such environment, crust will adsorb water from the environment. The additional free water in the crust will favor microbial spoilage of bread during storage.

### 2.7 Measurement of water reduction by weighing

The bread samples were weighed by a scale (XP 204, Mettler Toledo GmbH, Switzerland). The weight loss (in %) was calculated by

$$Weight\;loss=\frac{W_{day1}-W_{t}}{W_{day1}}\;100\%$$

where W_day1_ is the weight of the bread on day 1 and W_t_ is the weight of the bread on a certain measuring day. Measurements were performed in triplicate.

### 2.8 Texture analysis

Crumb firmness was measured in 6 replicates by uniaxial compression of a slice of bread crumb using a Texture Analyzer (TA.XT plus, Stable Micro Systems, United Kingdom). A bread slice (middle slice) with a thickness of 30 ​mm was compressed centrally with a Texture Analyzer. Prior to experiments, the Texture Analyzer was calibrated with a 5 ​kg load. An aluminum cylindrical probe with 100 ​mm length and 16 ​mm diameter was used to compress the sample to 40% of its original height at a speed of 1.70 ​mm/s. The trigger force was set to 5 ​g. The pre-test and post-test speeds were set to 1.00 ​mm/s. Crumb firmness is defined as the maximum force during the compression.

## 2.9 Light microscopy

The structure of different wax coatings was analyzed with a light microscope (DM6B, Leica Microsystems, Switzerland) using polarized light. 5% (w/w) of each wax was mixed in high oleic sunflower oil. The concentration of 5% was chosen to avoid the overlay of wax in order to ease the visualization. The sample preparation followed the procedure depicted by Blake et al. (2014): A drop of each molten wax coating solution was deposited onto a preheated glass microscope slide respectively. The molten wax solution was then pressed by a heated glass cover slip in order to ensure a thin sample (<1 ​mm) for the light microscopy. Afterwards, the slides were stored in a storage room with a constant temperature of 23 ​°C for 1 week before imaging in order to ensure a constant cooling rate of all wax samples.

### 2.10 X-ray micro-computed tomography (micro-CT) measurement

The micro-CT scanning was performed on Day 1, Day 4, and Day 14. To prepare the sample for the scanning, bread crust was separated carefully by hand with a knife. The crust samples were then inserted in the testing tubes of the CT Scanner (Scanco μCT 100) with a peak voltage of 55 ​kVp and a current of 109 ​μA. The image resolution was 7.4 ​μm. The image analysis was done with software Fiji (National Institutes of Health, US) and MITK GEM (National Institutes of Health, US).

### 2.11 Moisture sorption isotherm

The determination of moisture desorption isotherms was conducted using a gravimetric vapor sorption analyzer (SPSx-1 μ, ProUmid, Ulm, Germany). The measurements were performed in triplicate at a constant temperature of 23 ​°C. The tests started from a relative humidity at 90% and decreased progressively to 0% in 10% steps. The samples were considered to have reached equilibrium when the change in sample mass is less than 0.02% in 60 ​min.

### 2.12 Differential Scanning Calorimetry (DSC)

The melting and the crystallization behaviors of the wax coatings were analyzed with a Differential Scanning Calorimetry (DSC3+/500, Mettler Toledo GmbH, Schwerzenbach, Switzerland). Around 10–20 ​mg of the molten wax coating was weighed and filled into aluminum crucibles. The crucibles were then hermetically sealed. Prior to the measurements, the DSC device was calibrated with indium. Afterwards, the crucible containing the sample and the reference crucible were inserted into the furnace of the device. The starting temperature of each measurement was set to 25 ​°C, which was kept for 1 ​min. The sample was then heated up to 100 ​°C at a rate of 10 ​°C/min, followed by a cooling step from 100 ​°C to 20 ​°C at the same rate. A nitrogen flush of 50 cm3/min was used.

### 2.13 Statistical analysis

One-way analysis of variance (ANOVA) was evaluated with the help of OriginPro (2018) (OriginLab Inc., USA). A Tukey HSD (honestly significant difference) test was used to compare the statistical significance of treatment means at 5% significant level.

## 3 Results and discussion

### 3.1 Comparison of different coatings on bread staling

**Fig. 1:**
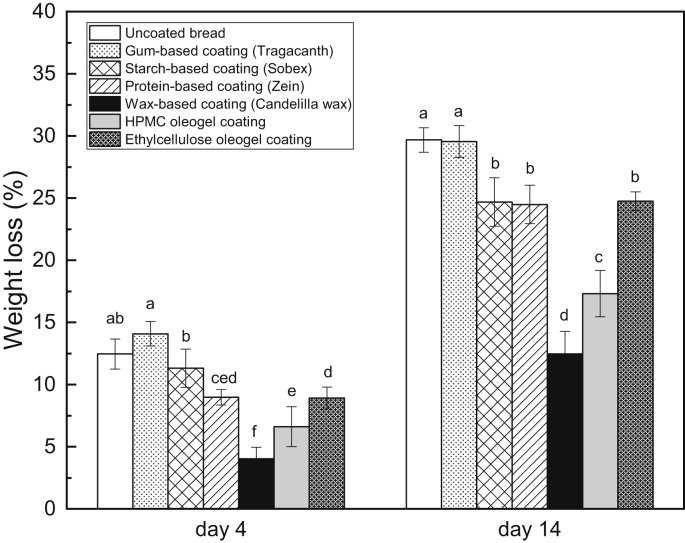
Weight loss of different coated breads on day 4 and day 14 (different letters on the same day indicate significantly different means at P ​≤ ​0.05).

Fig. 1 compares a variety of coatings with respect to the bread weight loss on day 4 and day 14 of the ambient storage. The weight loss reduction of the treated bread is believed to be one of the most relevant evaluations regarding the barrier effectiveness of a coating. Additionally, as all the fresh bread samples (on day 1) have an initial moisture content approximately 46% in the study, the weight loss is considered a good indicator of the change of moisture content of bread samples during storage. As illustrated in Fig. 1, the uncoated bread loses 12.5 ​± ​1.2% of its initial weight on day 4. Compared to the uncoated bread on day 4, the breads coated with the gum-based coating and the starch-based coating do not demonstrate significantly lower weight loss. However, the applications of the protein-based coating, the wax-based coating, the HPMC oleogel coating and the ethylcellulose olegel coating decrease bread weight loss significantly on day 4. After 14 days of storage, the weight loss of the uncoated bread further advances to 29.7 ​± ​1.0%. The bread coated with the gum-based coating shows similar results (29.5 ​± ​1.3% on day 14). On the other hand, all other coatings exhibit the ability to reduce moisture transfer significantly.

The gum-based coating made with tragacanth gum does not display any positive impact on the bread weight loss reduction on day 4 and day 14, which is likely caused by its hydrophilicity (Farahmandfar et al., 2017). It is also stated by Bertuzzi et al. (2007) that edible films and coatings consisting of plant gums are effective barriers to gas transport but present high water vapor permeability. With regard to the starch-based coating, the starch recrystallization occurred in the coating results in an increase in crystallinity of amylose and amylopectin (Matignon and Tecante, 2017), leading to a low affinity with water. According to Fu et al. (2015), amylopectin retrogradation is a slow process. Therefore, on day 4, amylopectin in the starch-based coating might only have retrograded slightly and a moderately lower value of weight loss compared to the control bread is observed. However, on day 14, amylopectin in the starch-based coating might have retrograded completely. Hence, a significant decrease in weight loss compared to the control bread is expected. Moreover, the protein-based coating, the wax-based coating as well as the oleogel coatings manifest satisfying moisture barrier properties, which might be due to their hydrophobicity.

**Fig. 2:**
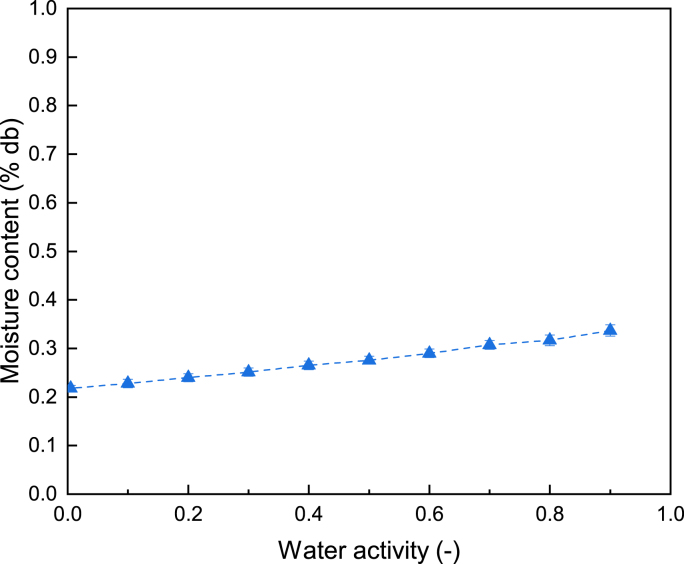
Moisture sorption isotherm of wax-based coating (20% candelilla wax).

It has been shown in the literature that hydrophobic barrier can limit moisture transfer in food products (Bourlieu et al., 2008). It should be noticed that the bread coated with the wax-based coating has the lowest weight loss on both day 4 and day 14. This is supported by the fact that coatings containing waxes can be used to reduce moisture loss and improve the surface appearance of various fruits and vegetables (Dhall, 2013). The excellent barrier efficiency of the wax-based coating could be attributed to two reasons: (1) Waxes normally are comprised of esters of fatty acids and long chain alcohols (Jannin and Cuppok, 2013). They do not possess any polar constituents nor hydrophilic parts, indicating that they cannot interact with water (Milovmovic and Picuric-Jovanevic, 2001). (2) Waxes have very low water vapor permeability, resulting in a slow moisture transfer (Spence et al., 2011). The water sorption isotherm of the wax-based coating depicted in Fig. 2, showcases that the equilibrium moisture content of the coating remains very low (below 0.35% (in dry basis)) within the water activity range of 0–0.9. Moreover, it can be seen from the graph that there is a moderate increase of the equilibrium moisture content with the elevating water activity, implying that the coating does not take up moisture markedly even when the relative humidity is rather high. Similar results were also observed by Bourlieu et al. (2008), who found that the moisture barrier containing wax presented an extremely low sorption of water with an equilibrium moisture content of 2.5% (in dry basis) at water activity of 0.98.

It is worth mentioning that the HPMC oleogel coating also exhibits good moisture barrier properties. The HPMC oleogel coating used in the study contains a high concentration of oil. The drying process included in the coating preparation procedure ensures the removal of water in the emulsion, which drives the oil droplets to be tightly packed in the polysaccharide network (Patel et al., 2014). Consequently, such coating could help reduce the transport of water owing to its low polarity. Even though the viscous gel texture of the HPMC oleogel coating makes it difficult to be evenly applied on the bread crust, the HPMC oleogel coating yields promising results regarding moisture loss retardation. Moreover, the ethylcellulose oleogel coating displays a moderate moisture barrier efficiency thanks to its hydrophobic nature. In addition, the semi-crystalline characteristics of ethylcellulose, which according to Davidovich-Pinhas et al. (2015) allow ethylcellulose to act as a gelling agent of liquid oils, facilitating the formation of oleogel.

**Fig. 3:**
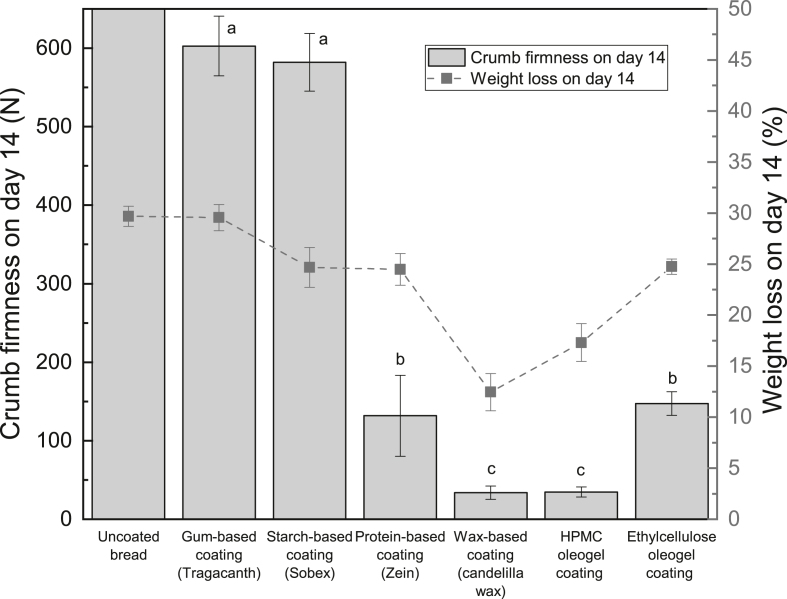
Crumb firmness of different coated breads on day 14 (different letters indicate significantly different means at P ​≤ ​0.05).

Fig. 3 reveals the crumb firmness of different coated breads on day 14. The crumb firmness of the standard bread (uncoated) on day 14 exceeds the measurement limit (616 ​N) of the Texture Analyzer. Similarly, the bread with the gum-based coating has a high firmness (around 600 ​N), most likely due to the large amount of moisture loss during ambient storage. The wax-coated bread presents the lowest crumb firmness owing to the lowest weight loss. Likewise, with a modest weight reduction, the bread with the HPMC oleogel coating also demonstrates a low crumb firmness. In addition, having similar weight loss, the protein-coated bread and the ethylcellulose oleogel-coated bread show comparable crumb firmness. In general, the correlation between the weight loss and the crumb firmness illustrated in Fig. 3 attests that crumb hardening could be reduced by lowering moisture loss of bread during ambient storage. It is also evident from the graph that by decreasing the bread weight loss from around 29.7% (uncoated bread on day 14) to about 24% (e.g. the protein-coated bread and the ethylcellulose oleogel-coated bread on day 14), the crumb firmness on day 14 can be reduced to approximately 150 ​N. When the weight loss is further decreased to roughly 17%, the crumb firmness is close to 35 ​N, which is close to values obtained for bread stored in plastic bag for 5 days (Gonzales-Barron et al., 2020). Hence, the obtained results highlight the potential of using coatings to retard bread staling.

### 3.2 Comparison of different wax-based coatings

As shown in the previous section, wax-based coatings are most effective in reducing bread moisture loss and crumb hardening during ambient storage compared to other coatings. Therefore, in this section, a number of waxes including beeswax, candelilla wax, rice bran wax, and carnauba wax are compared.

**Fig. 4:**
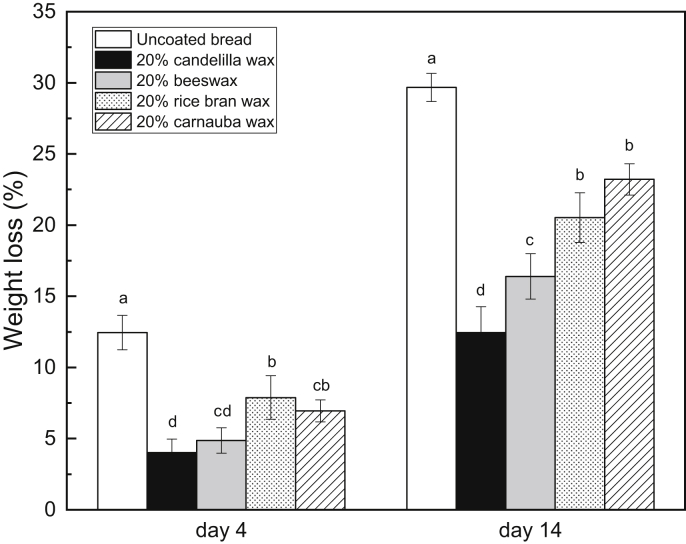
Weight loss of different wax-coated breads on day 4 and day 14 (different letters on the same day indicate significantly different means at P ​≤ ​0.05).

**Fig. 5:**
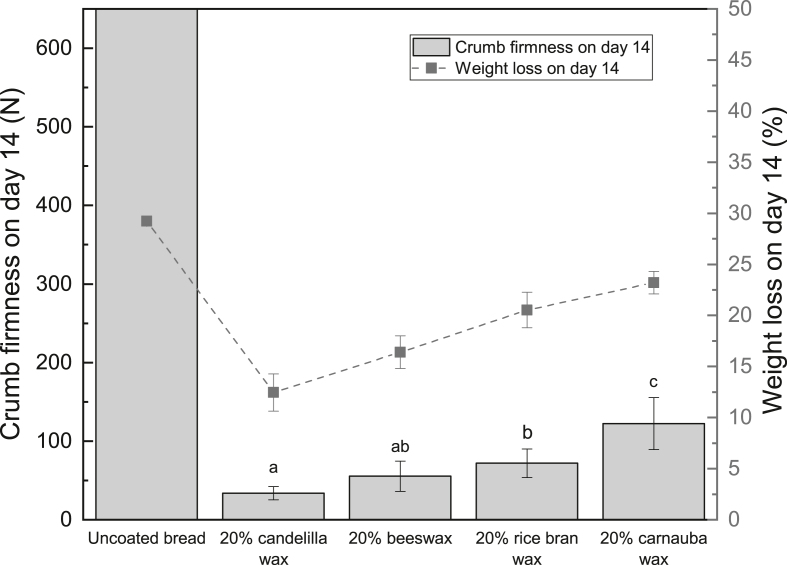
Firmness of different wax-coated breads on day 14 (different letters indicate significantly different means at P ​≤ ​0.05).

Fig. 4 depicts the weight loss of various wax-coated breads on day 4 and day 14. It is evident that all wax coatings can decrease the water loss in the bread significantly. In the short-term storage (4 days), the candelilla wax coating and the beeswax coating are most effective in reducing the bread weight loss from 12.5 ​± ​1.2% (uncoated bread on day 4) to 4.0 ​± ​0.9% and 4.9 ​± ​0.9% respectively. Additionally, the rice bran wax coating and the carnauba wax coating exhibit promising moisture barrier efficiency. In the long-term storage (14 days), the differences between each coating are more remarkable. The lowest weight loss (12.5 ​± ​1.8%) is seen in the candelilla wax-coated bread, followed by the beeswax-coated bread (16.4 ​± ​1.6%). The rice bran wax-coated bread and the carnauba wax-coated bread represent significantly higher weight loss compared to that of the candelilla wax-coated bread and the beeswax-coated bread. These observations are also reflected in the crumb firmness on day 14. As shown in Fig. 5, the candelilla wax-coated bread has the lowest crumb firmness, followed by the beeswax-coated bread. The carnauba wax-coated bread demonstrates the highest crumb firmness. The results again confirm that the crumb firmness is positively correlated to the bread weight loss.

**Fig. 6:**
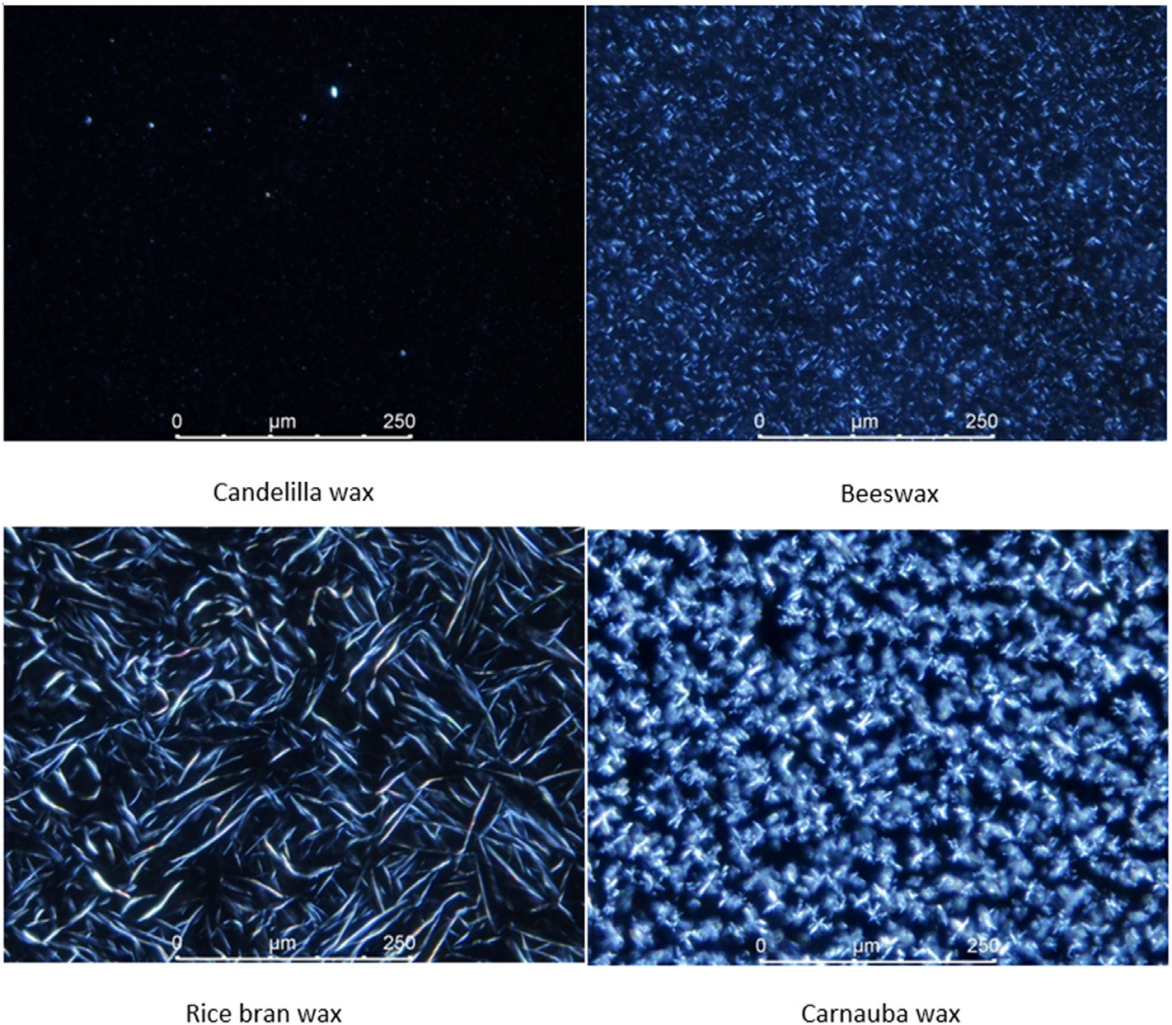
Polarized light microscopy images of different waxes.

The barrier efficiency of the wax coatings could be related to the wax structure and morphology, which are illustrated in Fig. 6. As demonstrated in the polarized light microscopy images, candelilla wax has very small crystals. This is supported by the findings from Blake et al. (2014), who revealed that candelilla wax exhibits grain-like crystals with a length between 3 and 5 ​μm, which also explains why they are hardly observed in our micrograph. Likewise, beeswax also demonstrates a small crystal size. On the other hand, rice bran wax displays needle-like crystals and carnauba wax contains dendritic crystals. Similar results were also reported by Blake et al. (2014). The dendritic morphology of carnauba wax might be one of the reasons why carnauba wax is least effective in reducing bread moisture loss compared to other waxes. According to Doan et al. (2017), dendritic crystals tend to interconnect and form a branched network with many voids, which could interfere with the network development of platelet crystals (Tavernier et al., 2017), resulting in the development of fissures and cracks in the coating, which indeed can be visible in the carnauba wax coating. The cracks will allow an easier and faster transport of water vapor (Spence et al., 2011), leading to a higher water vapor permeability of the coating. Furthermore, larger crystals will lead to a lower oil-binding capacity due to the smaller total surface areas (Blake et al., 2014), which is also responsible for the poorer performance of the rice bran wax coating and the carnauba wax coating in terms of reducing bread water loss during ambient storage. On the other side, fine crystals contribute to smooth coatings with less defects (e.g. pores, cracks, etc.) and to high oil binding capacities (Blake et al., 2014), giving rise to the greater moisture barrier efficiency of the candelilla wax coating and the beeswax coating.

**Fig. 7:**
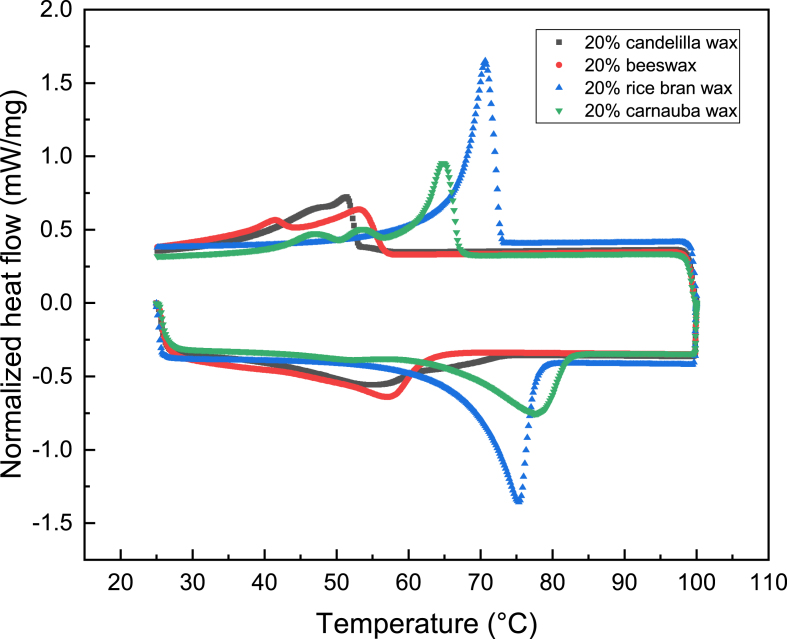
Differential scanning calorimetric thermograph of different wax-based coatings.

Moreover, the varying moisture barrier efficiencies of the wax coatings might be associated with their thermal properties. Fig. 7 presents the DSC thermograph of the wax-based coatings. Remarkable differences in the melting and crystallization behaviors of various wax coatings can be noticed. The highest peak meting temperature is seen in the rice bran wax coating, followed by the carnauba wax coating, the beeswax coating and the candelilla wax coating. The peak crystallization temperatures of different coatings decrease in the order of carnauba wax coating ​> ​rice bran wax coating ​> ​beeswax coating ​> ​candelilla wax coating. The high melting temperature of rice bran wax was also observed by Tavernier et al. (2017) and they attributed it to the presence of a high amount of long-chain wax esters. Carnauba wax is also reported to have a high melting temperature in some studies (Blake et al., 2014; Jannin and Cuppok, 2013). Upon cooling, high melting waxes crystalize first. In our study, the molten wax coating was applied on the hot bread surface (around 90 ​°C), which means the waxes with a higher crystallization temperature (i.e. carnauba wax and rice bran wax) have a shorter cooling time until crystallization starts, leading to a less organized and rougher network. As a consequence, a notable flakiness is observed in the carnauba wax coating and the rice bran wax coating. Besides, the carnauba wax coating possesses multiple melting peaks, indicating the existence of various chemical components with different thermal properties, which is in agreement with the study from Blake et al. (2014). The heterogeneous chemical composition would cause the formation of mixed crystals, which results in a high degree of crystalline disorder and eventually in forming a patchy as well as defected coating (Blake et al., 2014). As discussed earlier, the structural defects in the coating would favor water transport, which could explain the inferior moisture barrier properties of the carnauba wax coating.

### 3.3 Effects of crust treatments on water migration in breads

In this section, the enzymatic crust treatment and the chemical crust treatment are compared with the crust coating. Generally, bread crust contains a continuous protein phase and a discontinuous starch phase (Primo-Martin et al., 2007). Therefore, the crust structure can be modified by using enzymes or chemicals to change either protein phase or starch phase or both. It is believed that the porosity, hydrophobicity, tortuosity and other crust properties would be altered by modifying the crust structure, which might have impacts on water transport. Based on our preliminary studies, α-amylase and sodium hydroxide (NaOH) are selected to modify the bread crust, representing enzymatic crust treatment and chemical crust treatment respectively. α-amylase is assumed to play a role in the crust microstructure by hydrolyzing starch polymers, which would in turn have an influence on the moisture barrier properties of the crust. According to Altamirano-Fortoul et al. (2015), α-amylase can modify starch-protein interactions, posing effects on the water holding capacity and the cellular structure of the crust. NaOH is commonly used in the baking industry to make pretzel and is able to promote Maillard reaction during baking, causing alterations in the crust chemical structure, which might confer a higher moisture retention to the final product.

**Fig. 8:**
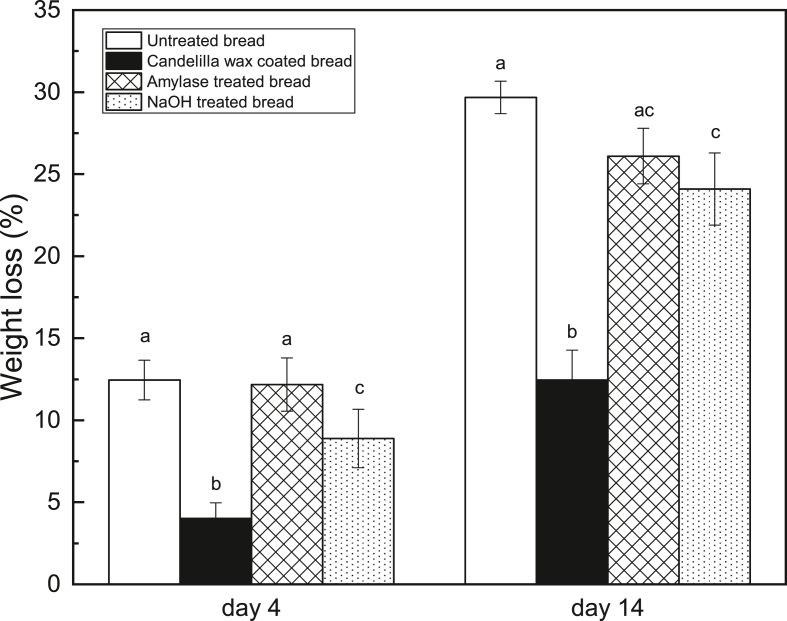
Weight loss of different treated breads on day 4 and day 14 (different letters on the same day indicate significantly different means at P ​≤ ​0.05).

Fig. 8 reveals the weight loss of different treated breads on day 4 and day 14. Once again, the candelilla wax coating is seen to be the most promising intervention in reducing bread moisture loss in both short-term and long-term storages, thanks to its low water vapor permeability. The amylase treated bread has a similar weight loss (12.2 ​± ​1.6%) to the untreated bread (12.5 ​± ​1.2%) on day 4 but a lower weight loss (26.1 ​± ​1.7%) on day 14 compared to the untreated bread (29.7 ​± ​1.0%). α-amylase can randomly hydrolyze α-1,4 linkages of starch, yielding low molecular weight sugars (Purhagen et al., 2011). As a result, the amylase treated crust might possess a disrupted structure, which could increase the tortuosity, hindering water transport during storage. On the other hand, the degradation of starch granules might result in pores in the crust, which would counter the positive effects of the increased tortuosity in terms of controlling moisture loss. Consequently, the amylase treatment does not display a significant reduction in the bread weight loss during ambient storage. Furthermore, NaOH treatment can significantly lower the bread weight loss compared to the untreated bread on both day 4 and day 14. NaOH is capable of hydrolyzing proteins in the crust into smaller peptides and amino acids, which will form inter-and intra-molecular bonds upon Maillard reaction during baking (Yao et al., 2006). Thus, the crust structure becomes denser and firmer due to the crosslinking, which would hamper the water migration.

It is noteworthy that even though the crust represents only a small percentage of the total length scale of bread, a variation in its moisture barrier properties could lead to significant impacts on water transport in bread during ambient storage. This is also in line with the study from Voogt et al. (2011), who reported that crust clearly influenced the moisture profile of the bread roll over time. Crust can function as a barrier for water migration and its barrier properties are dependent on the internal interactions between starch and proteins as well as the microstructure (Altamirano-Fortoul et al., 2015), which could explain the positive effects of the enzymatic and the chemical crust treatments on the bread weight loss during ambient storage.

Additionally, both the enzymatically treated bread and the chemically treated bread exhibit notably darker crust, most likely owing to the Maillard reaction. Altamirano-Fortoul et al. (2015) reported a similar observation in the enzymatically treated crust and concluded that the increased release of glucose from the enzymatic hydrolysis supplies additional glucose, which accelerates the Maillard reaction. In terms of the NaOH treated bread, in addition to the characteristic brown color, it possesses the distinct smoky odor of traditional pretzel.

**Fig. 9:**
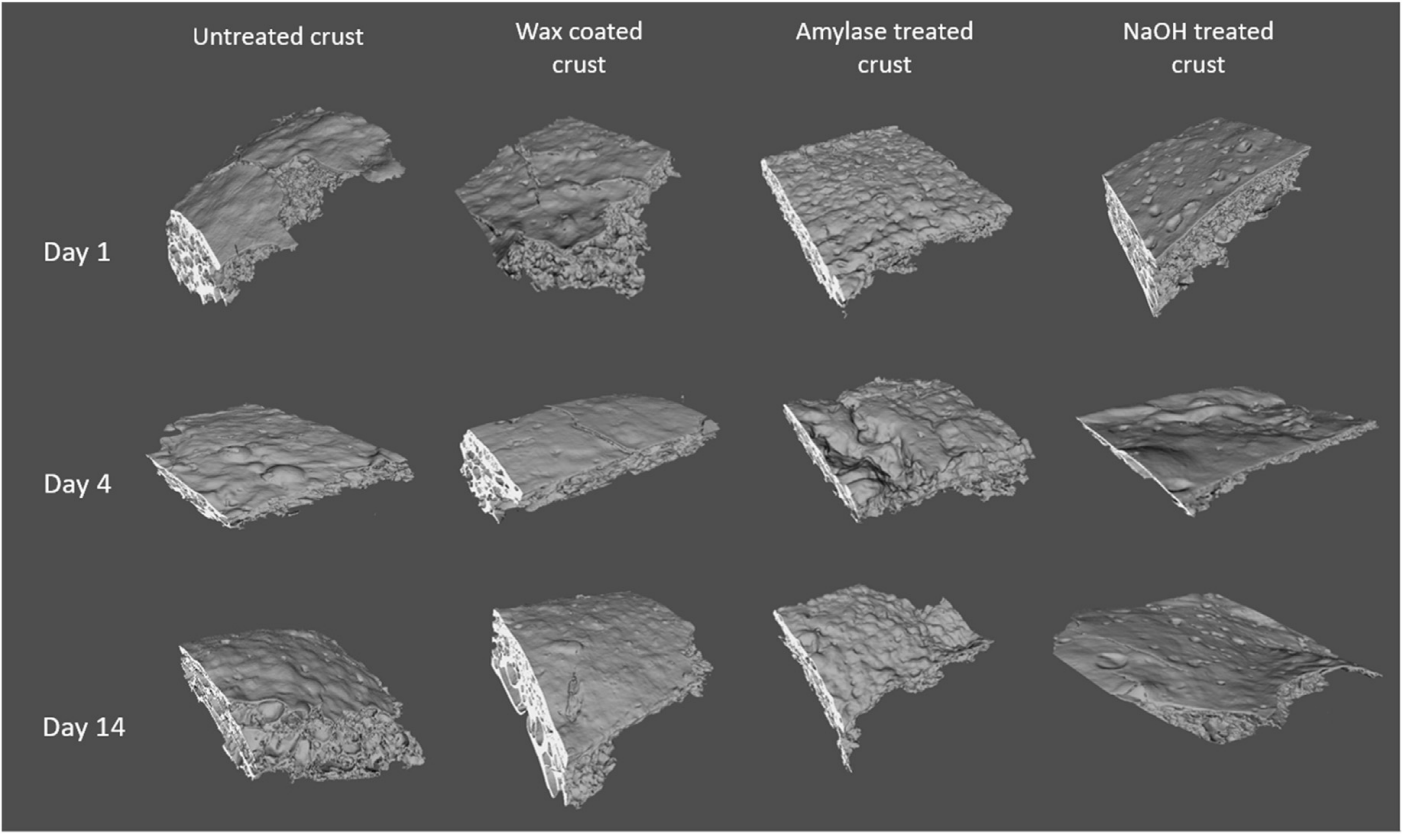
Crust morphology of different treated breads over time.

Fig. 9 presents the changes of the crust morphology of different treated breads with time. The amylase treated crust displays an irregular and uneven surface, presumably attributed to the starch degradation by the enzymatic hydrolysis. The NaOH treated crust shows a relatively rough surface with some dents/pits, possibly due to the protein hydrolysis and the deformation of gluten network. The untreated bread has a rather smooth surface on day 1, whereas, it becomes rough and uneven over time, which is likely caused by the volume shrinkage. On the other side, the wax coated crust remains fairly smooth during storage. It is plausible that the differences in the crust morphology contribute to the varying moisture barrier properties of the treated crusts, which is confirmed by the research conducted by Voogt et al. (2011), who demonstrated that the morphological parameters have a more profound impact on the water transport in bread than do material properties.

## 4 Conclusion

The efficacy of a number of bread coatings (gum-based coating, starch-based coating, protein-based coating, wax-based coating, HPMC oleogel coating, ethylcellulose oleogel coating) on minimizing water migration was compared. It was observed that the coatings containing hydrophobic substances achieved satisfying performance in retaining bread moisture during ambient storage, in particular when comparing the bread stored for five days in plastic bags (Gonzales-Barron et al., 2020). It is worth mentioning that the wax-based coating was most effective in reducing bread moisture loss and crumb hardening, thanks to its low affinity with water and low water vapor permeability. Additionally, the HPMC oleogel coating generated promising results, possibly owing to the high concentration of oil and its tightly packed structure. Several waxes including beeswax, candelilla wax, rice bran wax and carnauba wax were further investigated. Our results unveiled that all wax coatings could decrease bread moisture loss and crumb hardening significantly. The results further revealed that the 20% candelilla wax coating demonstrated the highest moisture barrier efficiency compared to other waxes, most likely attributed to its crystal morphology and thermal properties.

Various bread crust treatments, namely crust coating, enzymatic modification and chemical modification were studied. It was showed that the chemical modification and the enzymatic modification could alter the crust morphology and the crust moisture barrier properties, which would in turn have an impact on water transport in bread. However, they were less effective than the crust coating (i.e. 20 % candelilla wax coating) in terms of reducing bread water loss during ambient storage. With respect to wax coatings, hydrophobicity, microstructure (i.e. crystal size and crystal shape) and thermal properties of waxes are interconnected and all play important roles in moisture barrier efficiency of the coatings. Formation of cracks in certain wax coating (i.e. carnauba wax coating) could be related to crystal size and shape of the wax as well as thermal properties of the wax.

Based on our results, the candelilla wax coating beeswax wax coating are proved to have a high potential to retard bread staling. Furthermore, they could be applied as a water barrier in other high moisture foods. In addition, cellulose derivatives (e.g. HPMC oleogel coating) are good alternatives to waxes. However, further investigations are needed in order to improve the moisture barrier properties and texture of cellulose derivative-based coatings.

## CRediT authorship contribution statement

**Yi Chen:** Formal analysis, Data curation, Writing – original draft. **Theodoros Gavaliatsis:** Formal analysis, Data curation, Writing – original draft. **Simon Kuster:** Writing – original draft, Supervision. **Christian Städeli:** Writing – original draft, Supervision. **Peter Fischer:** Formal analysis, Data curation, Writing – original draft. **Erich J. Windhab:** Writing – original draft, Supervision.

## Declaration of competing interest

The authors declare that they have no known competing financial interests or personal relationships that could have appeared to influence the work reported in this paper.
